# Seroprevalence of *Helicobacter pylori* among dyspeptic patients in northern Lebanon: a 6-year retrospective study in two tertiary hospitals

**DOI:** 10.1099/acmi.0.000337

**Published:** 2022-04-27

**Authors:** Mohamad Bachar Ismail, Marwan Osman, Elie Bou Raad, Marcel Achkar, Monzer Hamze

**Affiliations:** ^1^​ Laboratoire Microbiologie, Santé et Environnement (LMSE), Doctoral School of Sciences and Technology, Faculty of Public Health, Lebanese University, Tripoli,, Lebanon; ^2^​ Faculty of Sciences, Lebanese University, Tripoli, Lebanon; ^3^​ Department of Public and Ecosystem Health, College of Veterinary Medicine, Cornell University, Ithaca, NY 14850, USA; ^4^​ Clinical Laboratory, El Youssef Hospital Center, Halba, Lebanon; ^5^​ Clinical Laboratory, Nini Hospital, Tripoli, Lebanon

**Keywords:** *Helicobacter pylori*, dyspepsia, IgG antibodies, epidemiology, serology, Lebanon

## Abstract

*
Helicobacter pylori* causes chronic gastritis and plays a significant role in duodenal/gastric ulcer disease and gastric cancer. Its prevalence varies among different populations and geographical areas. Here, in a hospital-based retrospective study, we investigated the seroprevalence of *H. pylori* infection in northern Lebanon. We examined the records of 4000 consecutive dyspeptic patients attending 2 tertiary care centres in the North (Tripoli) and Akkar (Halba) governorates. Seropositivity for *H. pylori* was determined using enzyme immunoassays investigating specific anti-* H. pylori* IgG antibodies. The association of infection with the available patients’ demographic characteristics was also evaluated. The mean age of our study population was 36.9±16.6 years. With 2486 female and 1514 male subjects, the overall female/male ratio was 1.64. In total, *H. pylori* seropositivity was detected in 1367/4000 (34.2 %) tested individuals. The multivariate logistic regression analysis showed that *H. pylori* infection is less prevalent in female than in male examined patients [adjusted odds ratio (OR): 0.84; 95 % confidence interval (CI): 0.73–0.96; *P*<0.013]. Seroprevalence gradually increased with age – from 14.6 % in patients below 18 years to 42.9 % in those above 49 years – and was significantly higher among Akkar patients compared to those from the North governorate: 49.6 versus 28.7 %, respectively (*P*<0.001). Overall, a third of symptomatic patients in northern Lebanon are infected with *H. pylori *. However, the prevalence of infection was markedly different in close geographical zones in this region. Additional screening studies using different screening methods are needed in the future to determine the accurate prevalence of this bacterium and its clinical implications to establish efficient national intervention strategies.

## Introduction

*Helicobacter pylori *, a motile spiral-shaped Gram-negative bacterium, is a highly successful human pathogen that has infected more than half of the world’s population [1]. First isolated in 1983, it colonizes the human stomach and is a well-established causative factor of peptic ulcers and chronic gastritis [2, 3]. Moreover, one decade after its identification, *H. pylori* was classified as a class I carcinogen [4] because of its important role in the pathogenesis of gastric malignancies, particularly gastric adenocarcinoma and gastric mucosa-associated lymphoid tissue lymphoma [5]. While *H. pylori* infection is common worldwide, its prevalence varies within and among countries, and this variability is linked to several factors, including geographical and socioeconomic aspects, ethnicity and age [6–8]. People residing in developing areas and facing poor socioeconomic conditions, including poverty, overcrowded living circumstances and inadequate sanitation/hygienic conditions, are at higher risk of infection [9].

*H. pylori* infections are normally acquired during childhood, but the exact transmission mode is poorly understood. Nevertheless, it is believed that person-to-person transmission routes such as oral–oral and faecal–oral transmission account for most cases of infection. However, only a minority of infected individuals develop *H. pylori *-associated gastrointestinal diseases, with most remaining asymptomatic [10]. Standard treatment for the successful eradication of this bacterial infection requires the administration of two or three antimicrobial agents (mainly clarithromycin, amoxicillin, levofloxacin and metronidazole), simultaneously or sequentially, combined with a proton pump inhibitor, histamine 2 blockers and bismuth-containing agents [11]. However, due to the steadily growing problem of antimicrobial resistance, the cure of *H. pylori* infection has become increasingly difficult [12]. For this reason, *H. pylori* was recently classified by the World Health Organization as a high-priority antibiotic-resistant bacterium that represents a great problem for public health. Globally, *H. pylori* is the single most important cause of infection-associated cancer. It accounts for >95 % of cases of gastric cancer, which, in turn, represents the third most common cause of cancer death worldwide. Consequently, the unclear antibiotic susceptibility patterns of this bacterium and the lower cure rates achieved with empirical therapies represent a global matter of concern.

At the diagnostic level, there are different screening methods for *H. pylori* infection. These include both invasive (e.g. gastrointestinal endoscopy and biopsy with subsequent rapid urease test, histological examination, culture and polymerase chain reaction) and non-invasive methods (e.g. serology, ^13^C urea breath test and stool antigen test) [13]. Most of the serological tests are designed to detect specific anti-* H. pylori* IgG antibodies in serum samples and are widely used to follow the epidemiological trends of *H. pylori* infection among different populations worldwide [8].

In Lebanon, there is no overall national estimate concerning *H. pylori* infection and recent data concerning its seroprevalence in the northern region of the country are scarce. Hence, the aims of this retrospective study were to assess the serological prevalence of *H. pylori* in a large sample of patients suffering from gastrointestinal (dyspeptic) symptoms in northern Lebanon and to evaluate the association between infection and the demographic parameters of the study population.

## Methods

### Study population

**Fig. 1.**
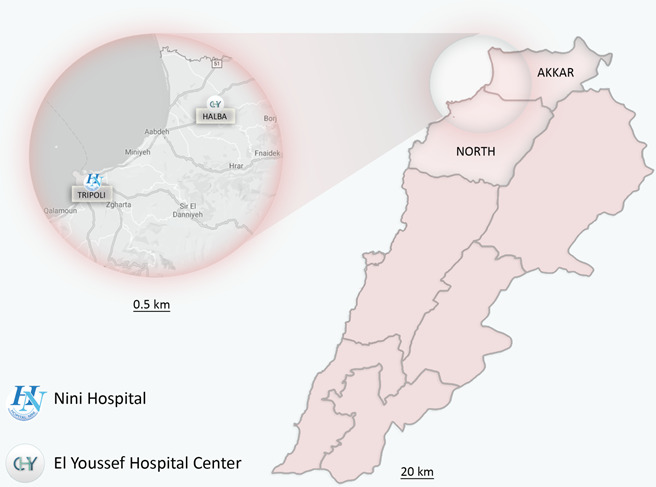
Geographical locations of the two tertiary care centres in the North and Akkar governorates in northern Lebanon.

This was a hospital-based retrospective study. In total, the study population consisted of 4000 consecutive individuals presenting various gastrointestinal symptoms and attending two large tertiary care centres – Nini hospital (*n*=2949) and El Youssef Hospital Center (*n*=1051) – as outpatients over a 6-year period (January 2013–December 2018). These two hospitals are respectively located in Tripoli and Halba, the central cities of an urban (North) and a rural (Akkar) governorate in northern Lebanon (Fig. 1).

### Sample collection and serological analysis

From each patient, 3 ml of venous blood were collected and centrifuged. Sera were immediately separated and tested. Information concerning the participant’s gender, age and residential area was also obtained at the time of sampling. Anti*-H. pylori* IgG antibodies were measured using different commercial immunoassays. Antibodies in sera from Nini Hospital were evaluated using two different kits: either a fully automated, solid-phase, two-step chemiluminescent enzyme immunoassay (IMMULITE/IMMULITE 1000 *H *. *pylori* IgG, Siemens) or the Orgentec anti-* H. pylori* IgG-ELISA (Orgentec Diagnostika GmbH). Serum IgG antibodies from El Youssef Hospital Center were measured using the NovaTec *pylori* IgG-ELISA kit (NovaLisa, NovaTec). According to manufacturers, the respective specificity and sensitivity of the IMMULITE immunoassay are 98.8 and 97 %, those of the Orgentec ELISA are 96.2 and 98.5% and those of the NovaLisa ELISA are 92 and 94.4 %.

### Statistical analysis

Data were analysed using R software (R Core team, version 4.1.0; R Studio, version 1.4.1106). Quantitative data were presented as the mean±standard deviation, and the categorical data were presented as frequency and associated proportions. The differences across groups were determined using the chi-squared test for categorical parameters. A multivariate logistic regression model was created. Seropositivity for *H. pylori* was the outcome, and the gender, age and geographical area were the explanatory variables. The tests were two-sided, with a type I error set at α=0.05.

## Results and discussion

In total, sera from 4000 patients from the North and Akkar governorates were evaluated in this study for the presence of anti-* H. pylori* IgG antibodies. The demographic characteristics of the study population are shown in Table 1. The overall seroprevalence of anti-* H. pylori* IgG antibodies was found to be 34.2 % (1367/4000) in our study. Although univariate analysis did not show an association between *H. pylori* infection and gender (*P*=0.098), the multivariate regression analysis revealed that female dyspeptic patients were significantly less likely than male patients to have *H. pylori* infection with an adjusted odds ratio (OR) of 0.84 [95 % confidence interval (CI): 0.73–0.96, *P*<0.013), after accounting for age and geographical area (Table 2). Moreover, age was significantly associated with seropositivity (*P*<0.001). Indeed, the infection prevalence increases gradually with age: from 14.6 % in subjects below 18 years (adjusted OR: 0.31; CI: 0.23–0.41; *P*<0.001) to 34.2 % in subjects aged 18–49 years (reference group) and reaching 42.9 % in those aged above 49 years (adjusted OR: 1.53; CI: 1.31–1.80; *P*<0.001). Importantly, seropositivity was significantly higher among Akkar patients (49.6 %) than the patients of the North governorate (28.7 %) (adjusted OR: 2.61; CI: 2.25–3.03; *P*<0.001).

**Table 1.** Demographic characteristics of the study population

	North patients	Akkar patients	Total
**No. and (%) of examined subjects**	2949 (73.7)	1051 (26.3)	4000 (100)
**No and (%) of females**	1793 (60.8)	693 (65.9)	2486 (62.1)
**No and (%) of males**	1156 (39.2)	358 (34.1)	1514 (37.9)
**Female/male ratio**	1.55	1.94	1.64
**Age range (years**)	1–97	1–86	1–97
**Mean age ±sd**	37.2±16.8	36.1±16	36.9±16.6

**Table 2.** Determinants of *Helicobacter pylori* seroprevalence, including gender, age and geographical area among dyspeptic patients in northern Lebanon using multivariate logistic regression analysis

		Descriptive analysis		Univariate analysis		Multivariate logistic regression analysis		
		Total	Seropositive, *n* (%)	*X* ^2^	*P*-value	adj. OR	95 % CI	*P*-value
**Gender**	Male*	2486	852 (34.3)					
	Female	1514	542 (35.8)	2.7	0.098	**0.84**	**0.73–0.96**	**0.013**
**Age**	<18	396	58 (14.6)			**0.31**	**0.23–0.41**	**<0.001**
	18–49*	2740	938 (34.2)					
	>49	864	371 (42.9)	**96.6**	**<0.001**	**1.53**	**1.31–1.80**	**<0.001**
**Geographical area**	Akkar*	1051	521 (49.6)					
	North	2949	846 (28.7)	**149.3**	**<0.001**	**0.38**	**0.33–0.44**	**<0.001**

*Reference group.

Continual assessment of the prevalence of *H. pylori* in both healthy and symptomatic individuals is important as it sheds light on the trends of this bacterial infection and helps in understanding the level and significance of its clinical implications in a given population. Indeed, *H. pylori* prevalence varies widely between different geographical areas and ethnic groups, and this is strongly linked to socioeconomic factors. In this context, two recent systematic reviews assessing the global prevalence of *H. pylori* infection showed an important variation among world zones and confirmed that infection rates in developing countries were higher than those in developed ones [6, 7]. For example, an *H. pylori* prevalence of 80 % or more was reported from several Latin American, African and Caribbean countries, while the prevalence was less than 20 % in several European areas. Notably, significant variability was also reported between distinct populations within the same continental area and even in the same country [8]. For example, while 80–90 % of Japanese individuals born before the 1950s were seropositive for *H. pylori *, the prevalence was found to be around 10 % and less than 2 % among subjects born around the 1990s and after the 2000s, respectively [14].

Recent data on the prevalence of *H. pylori* in Lebanon are scarce and only very few studies have specifically investigated its epidemiology in the northern region of the country (Table 3). In this study, we evaluated the serological prevalence of this bacterium in two northern governorates, the North and Akkar, in a large sample of 4000 dyspeptic patients presenting a variety of gastrointestinal disorders. Our results showed an overall seroprevalence of 34.2 % among this population.

**Table 3.** Prevalence of *Helicobacter pylori* in different healthy and dyspeptic populations in Lebanon

Year of publication	Detection method	Examined population	Sample size	Place/region of sample collection	Prevalence	Refs
**2000**	Modified urease technique	Adult symptomatic patients (≥20 years)	349	Hospital – North governorate	43.5 %	[15]
**2006**	Serology (IgG)	Adolescent students (14–18 years)	899	30 high schools scattered all over Lebanon	61.6 %	[16]
**2006**	Western bolt	Healthy adult blood donors (≥18 years)	104	Hospital – Beirut	68.3 %	[42]
**2007**	Stool antigen	Asymptomatic children (˂17 years)	414	Several Lebanese schools	21 %	[35]
**2012**	Serology (IgG)	Lebanese adults	308	Several Lebanese governorates	52 %	[17]
**2017**	Histological examination	Adult patients with dyspepsia (≥18 years)	294	Hospital – Zgharta, North governorate	52%	[19]
**2018**	^14^C urea breath test and histological examination	Adult patients with dyspepsia (≥18 years)	1030	Hospital – Sidon, South Lebanon	46.2%	[20]
**2021**	Stool antigens	Healthy children and adults	300	Two hospitals and one governmental medical clinic in Tripoli – North Lebanon	31 %	[42]
**2021**	Serology (IgG)	Patients with dyspepsia (1–97 years)	4000	Two hospitals – North and Akkar governorates	34.2 %	This study

In Lebanon, previous studies from 2006 showed that the prevalence of *H. pylori* among healthy adolescent and adult Lebanese individuals was 61.6 and 68.3 %, respectively [15, 16]. However, a cross-sectional study published 6 years later showed that this prevalence was 52 % in healthy adults [17]. This is in line with data reported in a systematic review showing that the prevalence of *H. pylori* was usually lower in the most recent surveys than those previously recorded in the same areas [18]. Notably, the two most recent Lebanese studies published in 2017 and 2018, examining symptomatic patients at two hospitals in the North and South of the country, reported respective *H. pylori* infection prevalences of 52 and 46.2 % [19, 20]. Thus, the overall *H. pylori* prevalence found in this study (34.2 %) is lower than those previously detected in dyspeptic patients in our country and even in most countries in Asia and the Middle East. However, our results are close to those found in symptomatic subjects in neighbouring Cyprus (39.8 %) [21] and in South Iran (31.2 %) [22].

A striking finding of our study is the significant difference in the infection prevalence between the two governorate patients. Indeed, while the infection prevalence among Akkar patients (49.6 %) is largely comparable to those reported in other national studies [19, 20], that of the North was unexpected as it revealed a strikingly low prevalence of infection (28.7 %) in tested symptomatic individuals. However, it is known that *H. pylori* prevalence varies widely even within the same country, and a significant difference in its prevalence among symptomatic patients living in different geographical zones of the same nation has been reported previously. For example, a study examining 3776 dyspeptic patients from various regions of Thailand revealed a marked difference in infection prevalences, with these ranging from 67.1 % in northern patients to only 32 % in those living in the southern peninsular region of the country [23]. In the same way, while very high rates of *H. pylori* infection were reported in India, a recent study examining symptomatic patients in Sikkim, a northeastern state of this country, found that only 27 % were infected [24]. Similar results were also found in the northwest region of Cameroon, where the infection prevalence reported among symptomatic patients attending four different hospitals was only 27.5 % [25]. One possible explanation for the difference between the prevalences in North and Akkar might be that the North governorate displays better socioeconomic, hygiene and living conditions compared to Akkar, which is one of the most deprived rural regions in Lebanon. Indeed, it is widely admitted that *H. pylori* infection occurs in significantly lower rates among residents in urban compared to rural sites. For example, Tadesse *et al.* [26] reported that infection among symptomatic Ethiopian patients was significantly lower in urban (28.8 %) compared to rural residents (71.2 %). However, the results in the North governorate remain striking, as they are considerably lower than others reported among patients from national regions with comparable socioeconomic and living conditions [19, 20]. It is possible that this difference in prevalence between the two governorates could partially be a result of the use of two different serological methods. Nevertheless, the sensitivity and specificity of the three serological tests used in this study are only slightly different and have a similar range of errors. Thus, no statistically significant differences related to diagnostic tools are probably expected to occur between these two groups.

In this study, the female/male ratio was 1.64. After accounting for age and geographical area, the multivariate regression analysis revealed that *H. pylori* infection is less prevalent in females compared to males in our examined patients. Previously, controversial results were documented concerning the association between gender and infection with *H. pylori *. Indeed, although rare studies showed higher prevalence in females [27, 28], the majority of reports and meta-analysis revealed, as in our case, a predominance of *H. pylori* infection among males [29, 30]. Nevertheless, several independent studies reported no statistically significant influence of gender on positivity rates [26, 31]. In this context, Zamani *et al.* [6] recently reported that although *H. pylori* infection is globally predominant in males, this predominance does not show statistical significance. Further, our results showed that infection with *H. pylori* was significantly associated with age and gradually increases with this (Table 2). Indeed, we found that only 14.6 % of subjects aged below 18 years were seropositive, but this percentage respectively increases to reach 34.2 and 42.9 % in patients aged 18–49 and above 49 years. These data are in line with reports showing that the rate of *H. pylori* infection is still increasing with age worldwide. Indeed, the global epidemiology of *H. pylori* infection in older individuals is characterized by significantly higher infection rates compared to younger subjects [6, 32–34]. Potential reasons for the decreased prevalence in younger generations may include the improvement across time of socio-economic status, lifestyle, living/residential environments, sanitary conditions and health behaviour. Of note, the prevalence we found in subjects aged below 18 years (14.6 %) is lower than that documented among asymptomatic individuals of the same age group in a previous Lebanese study showing a prevalence of 21 % [35]. Similarly, our results among adult patients showed lower prevalence compared to those reported nationally among both symptomatic and asymptomatic subjects (Table 3).

*H. pylori* is the primary cause of chronic gastritis and peptic ulcers and represents the strongest known risk factor for gastric malignancy. In Lebanon, Charafeddine *et al.* estimated that *H. pylori* infection currently results in half of the national gastric cancer cases [36]. Notably, the incidence of stomach cancer in Lebanon is increasing across time and is currently estimated to be 7.7 in females and 9.3 in males per year per 100 000 inhabitants [37]. Unfortunately, as recently reported, national gastric cancer cases are characterized by several aggressiveness features, including the high prevalence of diffuse-type histology and metastatic disease at diagnosis [38]. Importantly, recent prospective trials now confirm the positive role of *H. pylori* eradication in the reduction of human gastric cancer [39]. Moreover, the exploration of the possible introduction of population-based *H. pylori* screening and treatment programmes as a probable cost-effective strategy to prevent stomach cancer was recommended by the International Agency for Research on Cancer (IARC) [40]. In this context, Chen *et al.* [41] recently evaluated the population-based screen-and-treat strategy for *H. pylori* infection in Chinese asymptomatic general population and found it to be cheaper and more effective than the no-screen strategy for preventing gastric cancer, peptic ulcer disease and nonulcer dyspepsia. However, as both *H. pylori* and gastric cancer prevalences greatly differ globally, each country should evaluate the necessity of a national population-based *H. pylori* screening and treatment programme. Notably, it should be noted that in countries with a low incidence, the cost-effectiveness of such programmes might depend on the decrease of the burden of non-malignant gastric diseases [8]. Taken together, these data showed that in Lebanon a careful assessment of the significance of such a programme is required to evaluate its cost-effectiveness before national implementation.

*H. pylori* infection in patients enrolled in this study was diagnosed using serological assays. Due to their commercial availability, accuracy and low cost, serological tests are the most frequently used clinically to diagnose *H. pylori* infection. Moreover, unlike several other screening methods, their sensitivity is not affected by clinical conditions, such as the use of standard treatment, which normally leads to a low bacterial load in the stomach. Serology-based studies might thus represent the most cost-effective approach for large screening programmes and are therefore helpful in understanding the epidemiological aspects of *H. pylori* [8]. This, in turn, may help in planning and adopting effective prevention and intervention procedures. However, it is important to note that although serological assays possess several advantages, they are unable to discriminate between current and previous infections [8, 13]. Therefore, they are incapable of reliably reflecting the actual current epidemiological trend of *H. pylori* among an examined population. Consequently, to resolve this issue, some future national epidemiological studies should be realized with different diagnostic methods that ensure the presence of *H. pylori* at the diagnosis time (e.g. ^13^C urea breath test and histological examination).

In conclusion, we found that a third of dyspeptic Lebanese patients are seropositive for *H. pylori *, with a significant difference among individuals living in rural and urban regions. *H. pylori* infection prevalence was higher in males than in females and it increased gradually with age. In the future, additional multicentre prospective studies conducted at the national level and using different diagnostic tools are needed to confirm our findings.
